# Risk factors for contagious gastroenteritis in adult patients with diarrhoea in the emergency department - a prospective observational multicentre study

**DOI:** 10.1186/s12879-019-3754-4

**Published:** 2019-02-11

**Authors:** Florence Skyum, Court Pedersen, Vibeke Andersen, Ming Chen, Andreas Franke, Detlev Petersen, Wolfgang Ries, Christian Backer Mogensen

**Affiliations:** 1 Focused Research Unit in Emergency Medicine, Hospital of Southern Denmark, Kresten Philipsens Vej 15, DK-6200 Aabenraa, Denmark; 2 Focused Research Unit for Molecular Diagnostic and Clinical Research, Hospital of Southern Jutland, Kresten Philipsens Vej 15, DK-6200 Aabenraa, Denmark; 3 Department of Clinical Microbiology, Hospital of Southern Jutland, Sydvang 1, 6400 Sønderborg, DK Denmark; 4 Institute for Regional Health Research, University of Southern Denmark, JB Winsløw Vej 25, DK-5000 Odense, Denmark; 5 Institute of Molecular Medicine, University of Southern Denmark, JB Winsløw vej 25, DK-5000 Odense, Denmark; 6 Department of Infectious Disease, Odense University Hospital, JB Winsløws vej 4, DK-5000 Odense, Denmark; 7 Department of Laboratory- and Transfusionmedicine, Ev.Luth. Diakonissenanstalt zu Flensburg, Knuthstr.1, 24939 Flensburg, Germany; 8 Department of Medicine, Ev.Luth. Diakonissenanstalt zu Flensburg, Knuthstr.1, 24939 Flensburg, Germany; 9 Department of Medicine II, Malteser Krankenhaus St. Franziskus-Hospital, Waldstraße 17, 24939 Flensburg, Germany; 10 Focused Research Unit in Emergency Medicine, Hospital of Southern Jutland, Kresten Philipsens Vej 15, DK-6200 Aabenraa, Denmark

**Keywords:** Isolation procedures, Contact precautions, Norovirus, *Clostridium difficile*, Emergency department

## Abstract

**Background:**

Infectious gastroenteritis is common in the emergency department (ED). Patients infected with either Norovirus or toxigenic *Clostridium difficile* require special isolation procedures. The aims were to describe the aetiology of infectious gastroenteritis in the ED, evaluate whether current isolation procedures, based on clinical judgement are sufficient, and to identify information that might be used to identify patients requiring isolation.

**Methods:**

Prospective, observational, multicentre study. We collected information on symptoms, vital signs, travel history, the recent use of antibiotics, and infectious contacts and tested faecal samples for Norovirus, *C. difficile,* and enteropathogenic bacteria.

**Results:**

The study enrolled 227 patients, of whom 163 (71%) delivered a faecal sample for Norovirus analysis (13% positive), 171 (74%) for *C. difficile* (13% positive), and 173 (76%) for enteropathogenic bacteria (16% positive). In total 71% of the patients were isolated using strict precautions, 29% of the isolated patient and 14% of the patients who were not isolated had had a highly contagious GE. Risk factors for Norovirus included frequent vomiting (OR 5.5), recent admission of another patient with Norovirus (OR 2.6), and a short duration of diarrhoea. Risk factors for *C. difficile* infections included older age (OR 6.0), longer duration of diarrhoea (OR 5.2), mucus in stool (OR 3.5), and previous antibiotic use (OR 23.4).

**Conclusion:**

Highly contagious GE occurs in ¼ of the GE patients in the EDs, isolation based on clinical judgement is not very efficient. Several risk factors can predict the presence of Norovirus or toxigenic *Clostridium difficile*. It is uncertain whether this knowledge can improve isolation practices in ED settings.

**Trial registration:**

This study was retrospectively registered in the Clinical Trials Data Base (NCT02685527) and prospectively approved by the Regional Committees on Health Research Ethics for Southern Denmark (project ID S20140200) and Ethics Committee at the Medical Association of Schleswig-Holstein [“Ethikkommission bei der Ärztekammer Schleswig-Holstein”, project ID 120/15(I)] and registered with the Danish Data Protection Agency (project ID nr. 2008-58-0035/ 1608).

## Background

Acute gastroenteritis (GE) is a common reason for admission in emergency departments (ED) [1, 2]. Adult patients with Norovirus and toxigenic *Clostridium difficile* are prevalent in Northern Europe and require strict isolation, since they are highly contagious.

EDs are characterized by high patient volumes and a short duration of stay, and contact precautions (CP) are important for preventing the spread of contagious diseases to many other patients. According to international and national recommendations, patients with suspected Norovirus or *C. difficile* infection require a private room and toilet and health staff must wear personal protective equipment [3–5]. While the use of such isolation procedures minimizes the risk of disease spread, there are notable drawbacks since the isolation procedures demand more health-care resources, restrict patient mobility, decrease flexibility, and lead to less documented care and fewer physician visits [6–8].

At the time of admission there is a high degree of uncertainty with respect to GE aetiology, and the decision to initiate isolation is largely dependent on clinical judgement. It is, however, difficult to assess whether a patient has a contagious GE, based on clinical judgement and in a previous study we found that only one-quarter of the patients who were isolated had Norovirus or *C. difficile* infection [2].

Currently there are no evidence-based criteria for identifying the ED patients with acute GE due to Norovirus or *C. difficile*. The Kaplan criteria can be used to define Norovirus outbreaks [9], and the gastroenteritis severity score/Vesikari score can rate the severity of acute GE [10], but has no role in assessing whether a patient presenting to hospital with GE require isolation.

In 2012, a clinical prediction scale for hospital-acquired *C. difficile* infection was developed, but the scale was not designed for acutely admitted patients [11] and in 2014, a small Swedish study developed a risk score to predict viral gastroenteritis, but *C. difficile* were not included [12].

Biochemical markers have not been useful [11] and the results of stool samples can be delayed due to sampling and analysis time and are rarely available at the admission time when the decision of isolation precautions has to be made.

Thus, it is still unsettled whether a clinical evaluation, based on medical history and physical findings, can be used to distinguish GE caused by Norovirus or *C. difficile* infections requiring strict contact precautions from GE caused by less contagious pathogens, where no isolation is needed or if it possible to identify other information which might help the clinician to decide whether to isolate or not, before stool examination results are available.

The aims of the present study were to describe the aetiology of infectious gastroenteritis in an ED setting, to evaluate, if the current clinical judgment of need for strict contact precaution is able to identify the right patients, and to identify, if certain clinical information and findings can be used to identify patients with Norovirus or *C. difficile* infections and thus the need of strict contact precaution in the ED.

## Methods

### Study design and setting

We conducted a prospective, observational, multicentre study of adults acutely admitted to two university-affiliated hospital organizations in Southern Denmark (study period from the 1st April 2015 to the 31st August 2016) and Northern Germany (study period from the 15th November 2015 to the 31st July 2016). The hospital in Southern Denmark (Hospital of Southern Jutland) included two EDs, the hospital in Northern Germany included two hospitals, St. Franziskus Hospital and Diakonissenkrankenhaus in Flensburg.

Although the study sites were in two different countries, they were located in the same geographical region, with less than 50 km distance between the sites.

### Use of isolation procedures

The attending physicians made the decision on whether admitted patients required isolation procedures according to their individual judgement, whether they suspected Norovirus or *C. difficile* infections or not. This judgement was based on their general knowledge and experience with the diseases, as no national guidelines or local instructions existed concerning when to suspect these infections.

### Inclusion and exclusion criteria

We identified eligible patients by reviewing patients who were admitted to participating EDs during weekdays (Monday to Friday). Using electronic patient logistic systems, the participating EDs in Denmark were screened twice daily for eligible patients. At the Danish sites, the electronic patient logistic systems require that physicians specify a chief complaint. We reviewed patients with the following chief complaints: (i) “diarrhoea and/or vomit of presumed infectious genesis” or (ii) “abdominal pain” with the additional note “under observation for acute appendicitis”. We included this latter group as a previous study found that such patients are frequently discharged with a diagnosis of GE [2]. Fever is also a predefined chief complaint, but not used to identify patients with gastroenteritis in our setting.

We included patients over 18 years who met the standard definition of GE, namely: an acute onset of three or more loose stools or any vomiting in 24 h, excluding patients with (a) cancer of the bowel, irritable bowel syndrome, Crohn’s disease, ulcerative colitis, cystic fibrosis, coeliac disease, or other chronic illness characterized by symptoms of diarrhoea or vomiting, or (b) for whom symptoms were assumed to be due to drugs, alcohol, or pregnancy [13]. Furthermore we excluded patients who had an ileostomy or colostomy (stool counts are inaccurate), patients who were unable to communicate in Danish or German, and patients who were unable or unwilling to provide consent.

Once a patient was identified in the electronic patient logistic systems, the nurse caring for the patient was contacted by a member of the study team to clarify if the patient met inclusion criteria. If they did then a member of the research team visited the patient to obtain informed consent and conduct a bedside interview.

The German sites did not register patients in a readily accessible registration system. Instead, these sites were called by phone once daily to screen for possible study participants. All nurses in the participating EDs received general information on the project.

The research team consisted of three study nurses and the first author. The study nurses were native language speakers according to the county where they worked. Members of the research team instructed the nurses who were providing patient care to obtain and send three faecal samples (one for each of the examinations for Norovirus, *C. difficile* and enteropathogenic bacteria) from a single bowel movement. A standardized method for collecting stool samples was used in the participating hospitals. Samples were collected in a sterile specimen container, immediately refrigerated, and then transported to the microbiological department at Soenderborg Hospital, where they were analysed for Norovirus and *C. difficile* (PCR analysis by BD Max, Becton Dickinson, New Jersey, USA). All stool samples were tested for *C. difficile* Toxin B, and if positive were further analysed for the presence of the binary toxin. The analyses for enteropathogenic bacteria, by culturing stool specimens on agar plates, were done in local microbiology departments.

### Variables/ data sources

A standardized electronic questionnaire was used to obtain demographic data (patient identification number, date of admission, age, sex, medication, and comorbidities) and potential risk factors for infection. The questionnaire was available in German and Danish and the translation was conducted by native speakers of both languages. The questionnaire had been validated in a previous project [2]. The questionnaire was completed by research team members. Obtaining informed consent and interviewing the patient were performed in the same way in the Danish and German sites apart for the respectively spoken languages. Information on vital signs and isolation regimes was obtained from each patient’s medical file. Information on medications and comorbidities was self-reported.

We collected information on symptoms (duration of diarrhoea, number episodes of bowel movements on the day of symptom onset and number of episodes of vomiting on the day of symptom onset, faecal characteristics, and the presence of abdominal pain), vital signs including temperature, travel history, the recent use of drugs (including antibiotics), and infectious contacts, travel in the 2 months prior to the admission and current use of medications, recent use of antibiotics was defined as taking antibiotics on the day of admission, whereas the previous use of antibiotics was classified based on whether patients had completed an antibiotic treatment in the week prior to admission, in the month prior to admission, or in the 2 months prior to admission. In case of long term antibiotic use, the date of completing the cure was relevant. Data for “appearance of another patient in the ED with a positive sample for Norovirus or *C. difficile* within the last week*”* was generated using information contained in the study database.

The following three outcome variables were recorded: results of the faecal samples analyses for Norovirus, for *C. difficile,* and for enteropathogenic bacteria (*Salmonella* species, *Campylobacter* species, *Yersinia* species, and *Shigella* species).

### Study size

In a previous study from the Hospital of Southern Jutland returned 64% of the patients discharged with a gastroenteritis diagnosis a faecal sample, of which 20% were positive for Norovirus or *C. difficile* [2].

Analysing with logistic regression methods requires a minimum 50 patients and an additional 10 cases per analysed variable [14]. Since for practical reasons we limited patient enrolment to weekdays (excluding holidays), we expected to identify no more than 25 positive stool samples during a one-year study period at the two Danish sites. We therefore established study duration of 18 months in the Danish sites and 6 months in the German sites hospital, with an aim to have at least 50 cases in total. We predefined, in case of less than 50 cases, only a univariate analysis would be reported.

### Statistical methods

Questionnaires and laboratory results were collected electronically, stored according to the requirements of the Danish Data Protection Agency, and then merged and analysed in cooperation with a statistician. Data analysis was performed with Stata statistical software, version 14.2 (Stata Corp, College Station, Texas, USA). Continuous variables were described with median and interquartile range. Categorical variables were presented as absolute numbers and proportions. Independent risk factors were identified by logistic regression analysis and expressed in Odd Ratios (OR). t. Variables were treated as missing if clinical data or the results of faecal samples were unavailable. The OR for Norovirus and *C. difficile* were calculated separately, since these infections have distinct risk factors.

## Results

In total, the study included 227 patients, 198 (87%) in Denmark and 29 (13%) in Germany. 54% of the patients were female. The age group 18–44 years included 23% of the patients with a median age of 62 years.

**Fig. 1:**
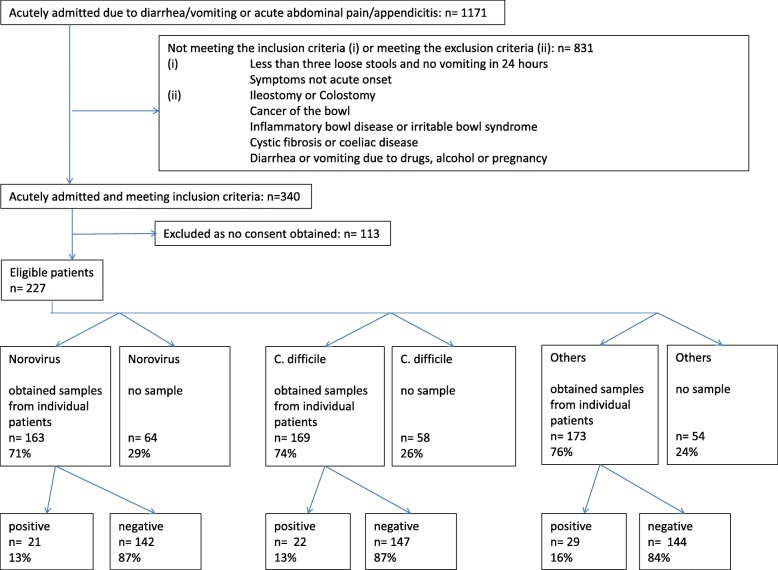
Patient flow

Of all included patients, 163 (71%) submitted a faecal sample for Norovirus analysis, 171 (74%) for *C. difficile* analysis, and 173 (76%) for enteropathogenic bacteria. Figure 1 outlines the number of patients in the study and the number of samples.

### Aetiology of gastroenteritis

Among the patients who delivered a faecal sample, 42% tested positive for a pathogenic microorganism, 13% had Norovirus, 13% *C. difficile,* and 16% enteropathogenic bacteria: 3% for a *Salmonella species* and 13% a *Campylobacter species*. Neither *Yersinia species* nor *Shigella species* were detected in the samples. Two patients with *Campylobacter* had concomitant infection, one with Norovirus and one with *C. difficile*. Patient characteristics in relation to the outcome of faecal examination are shown in Table 1.

**Table 1:** Results of the faecal sample examination

	Norovirus			*C. difficile*			Others (1)			No patogene (2)		
	Number of patients	Positive		Number of patients	Positive		Number of patients	Positive		Number of patients	Negative	
	n	n	%	n	n	%	n	n	%	n	n	%
Total	163	21	13	171	22	13	173	29	16	148	89	60
Sex												
Female	92	3	3	94	13	14	98	14	14	83	57	69
Male	71	18	25	77	9	12	75	15	19	65	32	49
Age (years)												
17–45	35	8	23	38	2	5	40	16	40	33	13	39
46–65	34	3	9	34	2	6	35	7	19	32	20	63
66–75	41	5	12	40	4	10	40	5	12	35	24	69
76–99	53	5	9	56	14	25	58	1	2	48	32	67
Medical history												
Duration of diarrhoea												
1 day	41	15	37	41	2	5	40	2	5	36	20	56
2–3 days	48	4	8	49	2	4	50	16	32	44	26	59
> 3 days	69	2	3	76	16	21	79	9	11	65	42	65
Number of defaecations on day of onset												
1–3 defaecations/day	42	4	10	43	3	7	44	5	11	39	28	72
4–10 defaecations/day	79	10	13	83	17	20	84	10	12	72	42	58
> 10 defaecations/day	36	7	19	37	1	3	39	14	36	32	15	47
Duration of vomiting												
No vomiting	58	7	12	59	10	17	65	19	29	54	24	44
1 day	45	12	27	49	1	2	46	4	9	40	28	70
> 1 day	55	2	4	57	8	14	59	6	10	51	37	73
Number of vomits on day of onset												
No vomiting	61	6	10	64	9	14	70	19	27	57	27	47
1–3 vomits	40	2	5	43	7	16	46	6	13	37	27	73
4–10 vomits	30	5	17	31	2	6	28	1	4	25	19	76
> 10 vomits	16	6	38	16	0	0	15	0	0	15	9	60
Mucus in stools												
No mucus	70	7	10	73	5	7	82	12	15	66	43	65
Patient reports mucus prior to admission	31	3	10	34	7	21	34	7	21	28	17	61
Abdominal pain												
No pain	51	6	12	58	9	16	57	3	5	46	32	70
Presense of abdominal pain	109	15	14	109	12	11	114	26	23	100	56	56
Travel												
No travel activity	124	16	13	129	19	15	132	13	10	112	74	66
Travel within Northern Europe	13	3	23	14	1	7	14	4	29	13	6	46
Travel within Southern or Eastern Europe	14	1	7	14	1	7	12	7	58	12	3	25
Travel outside Europe	10	1	10	11	0	0	14	5	36	10	6	60
Antibiotic treatment												
No antibiotic treatment	112	19	17	113	4	4	116	26	22	101	62	61
Recently/at admission	14	1	7	16	6	38	15	0	0	13	7	54
One week previous admission	11	0	0	12	4	33	13	1	8	11	6	55
One month previous admission	12	1	8	13	6	46	13	1	8	10	4	40
Two months previous admission	4	0	0	4	0	0	5	1	20	4	3	75
Contact to others with GE symptoms												
No contact to others	132	15	11	140	19	14	142	21	15	121	76	63
Contact to others	21	5	24	21	0	0	22	5	23	19	11	58
Epidemic risk												
No other positive sample within the last week in the ED	115	11	10	131	18	14						
Other positive sample within the last week in the ED	47	10	21	39	4	10						
Vital signs												
Systolic blood pressure												
100–140 mmHg	84	18	21	114	12	11	115	23	20	97	55	57
> 140 mmHg	34	3	9	38	6	16	47	4	9	39	28	72
< 100 mmHg	9	0	0	10	3	30	10	2	20	10	5	50
Pulse												
60–100/min	92	18	20	120	11	9	125	23	18	103	62	60
> 100/ min	28	3	11	35	7	20	36	5	14	32	19	59
< 60/min	7	0	0	9	3	33	8	0	0	8	5	63
Temperature												
36.5–37.5 °C	72	6	8	77	11	14	81	11	14	66	43	65
37.5–38.5 °C	44	9	20	44	5	11	47	9	19	40	20	50
> 38.5 °C	21	3	14	22	1	5	21	8	38	20	10	50
< 36.5 °C	21	3	14	22	3	14	20	0	0	17	14	82

1 others: Salmonella and Campylabacter species, Yersinia and Shigella were not detected

2 patients with faecal sample examination for Norovirus, C. difficile and others

### Seasonal variation

**Fig. 2:**
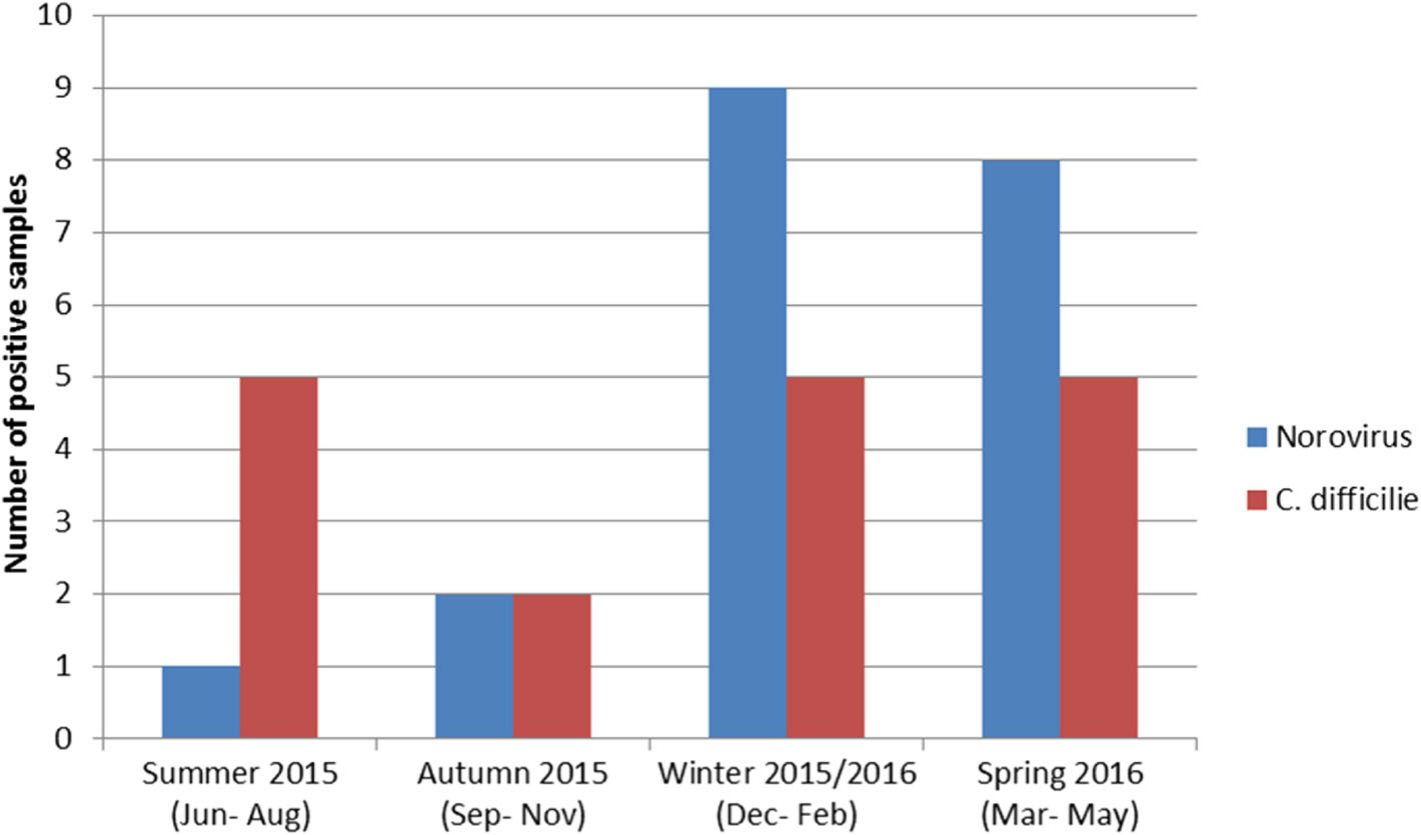
Seasonal variation of Norovirus and *C. difficile* infections

Seasonal variation for Norovirus and *C. difficile* infections is illustrated in Fig. 2*.*

The prevalence of Norovirus was highest in winter and spring. In contrast, there was no seasonal variation for *C. difficile* infections.

### Current use of isolation

Among the 172 patients who had faecal samples examined for both Norovirus and *C. difficile*, 42 (25%) tested positive for one or the other microorganism and 35 were placed in isolation. Among the 130 patients who tested negative for Norovirus and *C. difficile*, 87 were isolated. In total 71% of the patients were isolated.

The sensitivity to detect true contagious GE based on clinical judgement was thus 83% (95% CI 69–92%), the specificity 33% (26–42%), the positive predictive value 29% (21–37%), the negative predictive value 86% (74–93%) and the diagnostic accuracy 45% (38–53%).

### Risk factors for identification of patients with contagious GE

Table 2 presents the results of our analysis of potential risk factors for Norovirus and *C. difficile*.

**Table 2:** Risk factors for Norovirus or C. difficile infection

	Norovirus						C. difficile					
	Negative patients		Positive patients		OR	95% CI	Negative patients		Positive patients		OR	95% CI
	n	%	n	%			n	%	n	%		
Total	142	87	21	13			149	87	22	13		
Sex												
Female	89	97	3	3	1		81	87	13	14	1	
Male	53	75	18	25	**10**	2.8–36.5	68	89	9	12	0.8	0.3–2.0
Age (years)												
17–45	27	77	8	23	1		36	95	2	5	1	
46–65	31	91	3	9	0.3	0.1–1.4	32	94	2	6	1.1	0.1–8.5
66–75	36	92	5	13	0.4	0.1–1.4	36	90	4	10	2	0.3–11.6
76–99	48	91	5	9	0.4	0.1–1.1	42	75	14	25	**6**	1.3–28.2
Medical History												
Duration of diarrhoea												
1 day	26	63	15	37	1		39	95	2	5	1	
2–3 days	44	92	4	8	**0.2**	0.05–0.5	47	96	2	4	0.8	0.1–6.2
> 3 days	67	97	2	3	**0.05**	0.01–0.2	60	79	16	21	**5.2**	1.1–23.9
Number of defaecation on day of onset												
1–3 defaecations	38	90	4	10	1		40	93	3	7	1	
4–10 defaecations	69	87	10	13	1.4	0.4–4.7	66	80	17	20	3.4	0.9–12.5
> 10 defaecations	29	81	7	19	2.3	0.6–8.6	36	97	1	3	0.4	0.03–3.7
Duration of vomiting												
No vomitting	51	88	7	12	1		49	83	10	17	1	
1 day	33	73	12	27	2.6	0.9–7.4	48	98	1	2	**0.1**	0.01–0.8
> 1 day	53	96	2	4	0.3	0.05–1.4	49	86	8	14	0.8	0.3–2.2
Number of vomits on day of onset												
No vomitting	55	90	6	10	1		55	86	9	14	1	
1–3 vomtis	38	95	2	5	0.5	0.1–2.5	36	84	7	16	1.2	0.4–3.5
4–10 vomits	25	83	5	17	1.8	0.5–6.6	29	94	2	6	0.4	0.1–2.1
> 10 vomits	10	63	6	38	**5.5**	1.5–20.5	16	100	0	0	1 omitted	
Mucus in stools												
No mucus	63	90	7	10	1		68	93	5	7	1	
Patient reports mucus prior to admission	28	90	3	10	1.0	0.2–4.0	27	79	7	21	**3.5**	1.02–12.1
Adominal pain												
No pain	45	88	6	12	1		49	84	9	16	1	
Presense of abdominal pain	94	86	15	14	1.2	0.4–3.3	97	89	12	11	0.7	0.3–1.7
Travel												
No travel activity	108	87	16	13	1		110	85	19	15	1	
Travel within Northern Europe	10	77	3	23	2.0	0.5–8.1	13	93	1	7	0.4	0.1–3.6
Travel within Southern and Eastern Europe	13	93	1	7	0.5	0.1–4.2	13	93	1	7	0.4	0.1–3.6
Travel outside Europe	9	90	1	10	0.8	0.1–6.3	11	100	0	0	1 omitted	
Antibiotic Treatment												
No antibiotic treatment	93	83	19	17	1		109	96	4	4	1	
Recently/at admission	13	93	1	7	0.4	0.1–3.1	10	63	6	38	**16.4**	3.9–67.7
One week previous admission	11	100	0	0	1 omitted		8	67	4	33	**13.6**	2.8–64.9
One month previous admission	11	92	1	8	0.4	0.1–3.7	7	54	6	46	**23.4**	5.3–102.4
Two months previous admission	4	100	0	0	1 omitted		4	100	0	0	1 omitted	
Contact to others with GE symptoms												
No contact to others	117	89	15	11	1		121	86	19	14		
Contact to others	16	76	5	24	2.4	0.8–7.6	21	100	0	0	1 omitted	
Epidemic risk												
No other positive sample within the last week in the ED	104	90	11	10	1		113	86	18	14	1	
Other positive sample within the last week in the ED	37	79	10	21	**2.6**	1.0–6.5	35	90	4	10	0.7	0.2–2.3
Vital signs												
Systolic blood pressure												
100–140 mmHg	91	83	18	17	1		101	89	12	11	1	
> 140 mmHg	38	93	3	7	0.4	0.1–1.5	38	86	6	14	1.3	0.5–3.8
< 100 mmHg	10	100	0	0	1 omitted		7	70	3	30	3.6	0.8. 16
Pulse												
60–100/min	96	84	18	16	1		109	91	11	9	1	
> 100/ min	30	91	3	9	0.5	0.1–2.0	28	80	7	20	2.5	0.8–6.9
< 60/min	9	100	0	0	1 omitted		6	67	3	33	**4.9**	1.1–22.6
Temperature												
36.5–37.5 °C	66	92	6	8	1		66	86	11	14	1	
37.5–38.5 °C	35	80	9	20	2.8	0.9–8.5	39	89	5	11	0.7	0.2–2.3
> 38.5 °C	18	86	3	14	1.8	0.4–8.0	21	95	1	5	0.3	0.03–2.3
< 36.5 °C	18	86	3	14	1.8	0.4–8.0	19	86	3	14	0.9	0.2–3.7

*OR* Odds Ratio, *CI* Confidence Intervall

The following risk factors were significantly associated with Norovirus infection: male gender (OR 10), more than 10 episodes of vomiting on the day of symptom onset (OR 5.5), and the “appearance of another patient in the ED with a positive sample for Norovirus within the last week*”* (OR 2.6). A negative association was found with the duration of diarrhoea: two to 3 days (OR 0.2) or more than 3 days (OR 0.05).

A significant association for *C. difficile* was found for the following risk factors: 76–99 years of age (OR 6), pulse rate under 60/min (OR 4.9), more than 3 days of diarrhoea (OR 5.2), mucus in stools (OR 3.5), recent antibiotic treatment (OR 16.4), and antibiotic treatment completed either one week prior to admission (OR 13.6) or one month prior to admission (OR 23.4). A negative association was found with the duration of vomiting (OR 0.1).

Neither multivariate analyses nor risk score modelling were performed given the limited number of positive cases.

## Discussion

This study revealed three major findings: The results of the faecal samples (13% positive for Norovirus, 13% positive for *C. difficile*, and 16% had another identifiable enteropathogenic bacterium*)*, the ability to decide isolation criteria based on clinical judgement had a reasonable sensitivity of 83%, but a low specificity (33%) and accuracy (45%), and finally we confirmed well known risk factors for Norovirus (short duration, vomiting, other patients with Norovirus infection) and for *C. difficile* (longer duration, high age, recent antibiotic treatment, no vomiting).

Norovirus infections peaked in winter and spring, whereas in contrast there was no seasonal variation in the prevalence of *C. difficile* infections. Our findings concerning seasonal variation are similar to findings from an Australian study [18].

The identification rate of 42% for any stool pathogen mirrors results from other OECD countries as outlined in a 2013 review of the prevalence of gastrointestinal pathogens [15]. We presume that the different study periods do not give a difference in the aetiology. Our findings concerning the frequencies of *C. difficile* and Norovirus infections were comparable to results from the US [16–18]. *Salmonella* infections were found with similar frequencies, whereas *Campylobacter* infections were more frequent than in the US [19] and less frequent than in Germany [20].

Current isolation procedures, which are based on clinical judgement, resulted in the isolation of 71% of patients with GE symptoms. Current isolation practices appear consistent with findings from a 2014 study [2].

Well known risk factors for *C. difficile* include older age, the use of antibiotics, and hospitalization [21, 22]. One study looked at the following additional risk factors and found the most important to be antibiotic use, an overnight healthcare stay in the previous 3 months, and previous *C. difficile* infection [16]. The risk factors older age and previous antibiotic use were confirmed in our study. Furthermore we found additional possible risk indicators, specifically diarrhoea duration of more than 3 days and the presence of mucus in stool. The risk factor “low pulse rate” surprised for *C. difficile* infection. This infection mainly occurs in the patient groups of older age, and elderly do not always respond with tachycardia. Reasons for this might be electric heart diseases or treatment with Beta Blockers. A recent review investigates transmission pathways of *C. difficile,* where contact to symptomatic or asymptomatic carriers in the community setting is found a relevant source of infection [23]. In contrast, the risk factor “appearance of another patient in the ED with a positive sample for *C. difficile* within the last week*”* did not show significance in our study.

Known risk factors for Norovirus include contact with other individuals with gastroenteritis symptoms [9], treatment with Proton pump inhibitors [24] and belonging to high risk groups: young children, elderly, travellers, soldiers, and patients who are immunocompromised or have received an organ transplants [25]. Somewhat surprisingly, we found that male gender was a significant risk factor for Norovirus infection, although we did not identify a plausible explanation for this finding. Two other recent studies found no gender difference in the rate of infection for Norovirus infection [26, 27]. Thus, we suspect that our finding of gender differences is an incidental finding.

Contact with other individuals with symptoms of gastroenteritis did not have a significant association with Norovirus infection, neither in our study nor in a Swedish study [12]. There was, however, a significant association with the variable “appearance of another patient in the ED with a positive sample for Norovirus within the last week*”.* This likely reflects the fact that community-acquired Norovirus infections presents in out-breaks, but that transmission in most cases does not occur directly from a person recognized as contagious.

Our results need some considerations for clinical implication and further studies.

Around ¼ of all admitted patients to the ED has a very contagious GE which requires strict contact precautions but it is difficult to judge whom to isolate based on clinical judgement and it is a concern that the diagnostic accuracy is so low concerning isolations. Short duration, vomiting and similar cases in the department might aid to guide the clinician concerning norovirus infection, and high age and recent antibiotic treatment raises suspicion of *C. difficile* infection. However, we identified several factors that should be considered when evaluating the need for isolation in the ED. Further studies with a higher number of participants are needed to investigate whether these factors can be used to generate a simple, rapid, clinical-based scoring system that can assist front-line clinicians in making accurate patient isolation decisions. Of note, there is a likely need for two different risk scoring systems, one for Norovirus and another for *C. difficile*, since patients with these infections have distinct demographic characteristics and varied clinical presentations. Our data and previous publications indicate that developing such clinical rules will be challenging, and thus there is a need to explore other means of improving isolation procedures. Specifically, we believe that new rapid tests for faecal pathogens have an important role to play and should serve as a focus for future clinical studies. While we predict that such tests may not reduce the number of patients inappropriately placed in isolation, they may significantly reduce the time spend in isolation since time between obtaining a sample and the results can diminished.

The strengths of our study include systematic and prospective data collection in a relevant clinical context, an extended study period of 18 months, and a multicentre setting.

However, our study does have some limitations. Not all patients could deliver faecal samples, either because the diarrhoea resolved or they were discharged from hospital before their samples could be collected. While we do not believe that this had a major impact on our interpretation of the study data, we cannot exclude the possibility that patients with less severe disease had a different pathogen distribution. Specifically, it is plausible that such patients have a lower prevalence of *C. difficile*. Another study limitation is that approximately one-third of patients who met the inclusion criteria were not included in the study, in large part due to a failure to obtain consent. Thus, it is possible that elderly and the more fragile patients are under-represented in the study group. Since age and comorbidity are recognized risk factors for *C. difficile*, it is thus possible that the study underestimated the prevalence of *C. difficile*.

## Conclusion

Norovirus and *C. difficile* are common pathogens in patients admitted with acute gastroenteritis. There are several risk factors which can help identify these patients, but it is uncertain whether this knowledge can be used to improve the rational use of isolation in the ED. More studies in this area are warranted. Rapid diagnostics may be an alternative approach and are a potential future research avenue.

## Abbreviations

CI: Confidence Interval
CP: Contact Precautions
ED: Emergency Department
GE: Gastroenteritis
OECD: Organisation for EconomicCo-operation and Development
OR: Odds Ratio
US: United States

## References

[1] Mounts AW, Holman RC, Clarke MJ, Bresee JS, Glass RI (1999). Trends in hospitalizations associated with gastroenteritis among adults in the United States, 1979-1995. Epidemiol Infect.

[2] Skyum F, Abed OK, Mogensen CB (2014). Clinical information on admission is insufficient to determine the appropriate isolation regimen for acute gastroenteritis. Dan Med J.

[3] Siegel JD, Rhinehart E, Jackson M, Chiarello L (2007). 2007 guideline for isolation precautions: preventing transmission of infectious agents in health care settings. Am J Infect Control.

[4] Institut SSS (2016). Nationale Infektionshygiejniske Retningslinjer.om supplerende forholdsregler ved infektioner og bærertilstand i sundhedssektoren. Infektionshygiejne CEf.

[5] Stallmach. S2k Leitlinie - Gastrointestinale Infektionen und Morbus Whipple. In: e.V. AAdwmF, editor. https://www.awmf.org/uploads/tx_szleitlinien/021-024l_S2k_Infekti%C3%B6se_Gastritis_2015-02-verlaengert.pdf.

[6] Morgan DJ, Pineles L, Shardell M, Graham MM, Mohammadi S, Forrest GN (2013). The effect of contact precautions on healthcare worker activity in acute care hospitals. Infect Control Hosp Epidemiol.

[7] Stelfox HT, Bates DW, Redelmeier DA (2003). Safety of patients isolated for infection control. JAMA.

[8] Evans HL, Shaffer MM, Hughes MG, Smith RL, Chong TW, Raymond DP (2003). Contact isolation in surgical patients: a barrier to care?. Surgery.

[9] Kaplan JE, Feldman R, Campbell DS, Lookabaugh C, Gary GW (1982). The frequency of a Norwalk-like pattern of illness in outbreaks of acute gastroenteritis. Am J Public Health.

[10] Freedman SB, Eltorky M, Gorelick M (2010). Evaluation of a gastroenteritis severity score for use in outpatient settings. Pediatrics.

[11] Chandra S, Latt N, Jariwala U, Palabindala V, Thapa R, Alamelumangapuram CB (2012). A cohort study for derivation and validation of a clinical prediction scale for hospital-onset Clostridium difficile infection. Can J Gastroenterol.

[12] Andreasson T, Gustavsson L, Lindh M, Bergbrant IM, Raner C, Ahrén C (2014). Evaluation of anamnestic criteria for the identification of patients with acute community onset viral gastroenteritis in the emergency department--a prospective observational study. Scand J Infect Dis.

[13] Majowicz SE, Hall G, Scallan E, Adak GK, Gauci C, Jones TF (2008). A common, symptom-based case definition for gastroenteritis. Epidemiol Infect.

[14] Green SB (1991). How many subjects does it take to do a regression analysis. Multivar Behav Res.

[15] Fletcher SM, McLaws ML, Ellis JT (2013). Prevalence of gastrointestinal pathogens in developed and developing countries: systematic review and meta-analysis. J Public Health Res.

[16] Abrahamian FM, Talan DA, Krishnadasan A, Citron DM, Paulick AL, Anderson LJ, et al. Clostridium difficile infection among US emergency department patients with diarrhea and no vomiting. Ann Emerg Med. 2017;70(1):19–27.e4. 10.1016/j.annemergmed.2016.12.013. Epub 2017 Feb 24 10.1016/j.annemergmed.2016.12.013 28242058

[17] Patel MM, Widdowson MA, Glass RI, Akazawa K, Vinjé J, Parashar UD (2008). Systematic literature review of role of noroviruses in sporadic gastroenteritis. Emerg Infect Dis.

[18] Cardemil CV, Parashar UD, Hall AJ. Norovirus infection in older adults. Epidemiology, Risk Factors, and Opportunities for Prevention and Control. Infect Dis Clin North Am. 2017;31(4):839–870. 10.1016/j.idc.2017.07.012. Epub 2017 Sep 12. 10.1016/j.idc.2017.07.012 PMC6546097 28911830

[19] Bresee JS, Marcus R, Venezia RA, Keene WE, Morse D, Thanassi M (2012). The etiology of severe acute gastroenteritis among adults visiting emergency departments in the United States. J Infect Dis.

[20] Jansen A, Stark K, Kunkel J, Schreier E, Ignatius R, Liesenfeld O (2008). Aetiology of community-acquired, acute gastroenteritis in hospitalised adults: a prospective cohort study. BMC Infect Dis.

[21] Bartlett JG (2006). Narrative review: the new epidemic of Clostridium difficile-associated enteric disease. Ann Intern Med.

[22] Tilton CS, Johnson SW. Development of a risk prediction model for hospital-onset Clostridium difficile infection in patients receiving systemic antibiotics. Am J Infect Control. 2018(18)30872–1. 10.1016/j.ajic.2018.08.021. [Epub ahead of print] 10.1016/j.ajic.2018.08.021 30318399

[23] Durovic A, Widmer A, Tschudin Sutter S. New insights into transmission of Clostridium difficile infection - a narrative review. Clin Microbiol Infect. 2018;24(5):483–492. 10.1016/j.cmi.2018.01.027. Epub 2018 Feb 7. Review 10.1016/j.cmi.2018.01.027 29427800

[24] Prag C, Prag M, Fredlund H (2017). Proton pump inhibitors as a risk factor for norovirus infection. Epidemiol Infect.

[25] Glass RI, Parashar UD, Estes MK (2009). Norovirus gastroenteritis. N Engl J Med.

[26] Skyum F, Andersen V, Chen M, Pedersen C, Mogensen CB. Infectious gastroenteritis and the need for strict contact precaution procedures in adults presenting to the emergency department: a Danish register-based study. J Hosp Infect. 2018;98(4):391–397. 10.1016/j.jhin.2017.11.001. Epub 2017 Nov 8. 10.1016/j.jhin.2017.11.001 29128345

[27] Bernard H, Höhne M, Niendorf S, Altmann D, Stark K. Epidemiology of norovirus gastroenteritis in Germany 2001-2009: eight seasons of routine surveillance. Epidemiol Infect. 2014;142(1):63–74. 10.1017/S0950268813000435. Epub 2013 Mar 21. 10.1017/S0950268813000435 PMC9152553 23517686

